# Classification of multiple sclerosis women with voiding dysfunction using machine learning: Is functional connectivity or structural connectivity a better predictor?

**DOI:** 10.1002/bco2.217

**Published:** 2023-01-28

**Authors:** Khue Tran, Betsy H. Salazar, Timothy B. Boone, Rose Khavari, Christof Karmonik

**Affiliations:** ^1^ EnMed Program Texas A&M School of Engineering Medicine Houston Texas USA; ^2^ Department of Urology Houston Methodist Hospital Houston Texas USA; ^3^ Translational Imaging Center Houston Methodist Research Institute Houston Texas USA

**Keywords:** brain connectivity, functional MRI, machine learning, multiple sclerosis, neurogenic bladder, voiding dysfunction

## Abstract

**Introduction:**

Machine learning (ML) is an established technique that uses sets of training data to develop algorithms and perform data classification without using human intervention/supervision. This study aims to determine how functional and anatomical brain connectivity (FC and SC) data can be used to classify voiding dysfunction (VD) in female MS patients using ML.

**Methods:**

Twenty‐seven ambulatory MS individuals with lower urinary tract dysfunction were recruited and divided into two groups (Group 1: voiders [V, *n* = 14]; Group 2: VD [*n* = 13]). All patients underwent concurrent functional MRI/urodynamics testing.

**Results:**

Best‐performing ML algorithms, with highest area under the curve (AUC), were partial least squares (PLS, AUC = 0.86) using FC alone and random forest (RF) when using SC alone (AUC = 0.93) and combined (AUC = 0.96) as inputs. Our results show 10 predictors with the highest AUC values were associated with FC, indicating that although white matter was affected, new connections may have formed to preserve voiding initiation.

**Conclusions:**

MS patients with and without VD exhibit distinct brain connectivity patterns when performing a voiding task. Our results demonstrate FC (grey matter) is of higher importance than SC (white matter) for this classification. Knowledge of these centres may help us further phenotype patients to appropriate centrally focused treatments in the future.

## 1 INTRODUCTION

Multiple sclerosis (MS) is an autoimmune inflammatory disease where axons in the central nervous system (CNS) are demyelinated, leading to a plethora of symptoms, including neurogenic voiding dysfunction (VD), characterized by urinary retention or incomplete bladder emptying.^1^, ^2^ If left untreated, VD can lead to serious sequela, including urinary tract infections, sepsis, bladder or kidney stones and ultimately permanent renal failure.^1^, ^3^ Currently, the only treatment with moderate effectiveness for VD is self‐catheterization, which can be a significant burden for MS patients with or compromised dexterity or spasms.^1^

The advancement of neuroimaging techniques over the past few decades has allowed us to investigate the underlying neurological mechanism of neurogenic lower urinary tract dysfunction (NLUTD) in MS patients, specifically the connections between different brain regions in the presence of disrupted neuronal signals, including functional connectivity (FC) and structural connectivity (SC).^4^ FC is based on the notion that brain regions exhibiting similar activations (synchronized) during a time course share the same information and are therefore said to be connected in a functional manner.^5^ Meanwhile, SC refers to the white matter tracts physically linking a variety of brain regions.^5^ Previous studies have demonstrated both FC and SC are affected in MS patients with NLUTD, where CNS axons are demyelinated and thus disrupt neuronal signal transduction.^6^, ^7^, ^8^ Although the CNS has the innate ability to drive remyelination of disrupted axons, it is only partial, leading to inefficient recovery of function.^9^, ^10^ Often overlooked is the difference in FC and SC of MS patients with VD compared with those without. This raises the question of whether FC and SC can be used as predictors for VD in MS patients. Here, we applied novel machine learning techniques to address this very topic.

Machine learning (ML) is a method of data evaluation that uses datasets with known outcomes to train a computer algorithm to perform an assessment on new data without explicit human interaction. Recently, there have been a growing number of ML applications in characterizing brain connectivity measures, including assessing disease severity and symptoms^11^, ^12^ and predicting disease courses,^13^ as well as the patient response to treatment.^14^ With the growing potential applicability of ML, in this study, we sought to investigate the validity of using ML to classify MS patients into voiders or VD groups, utilizing FC and SC, both separately and combined, as predictors.

## 2 METHODS

### 2.1 Subjects

The study and its ethical code were approved by the Houston Methodist Institutional Review Board (IRB). Informed consents were signed by all participants prior to enrolment. Adult females (≥18 years old) with at least 6 months of clinically stable MS [Expanded Disability Status Score (EDSS) ≤ 6.5] and at least 3 months of symptomatic NLUTD (including both storage and voiding symptoms) were referred to our fellowship‐trained neurourologists at the Houston Methodist Neurourology clinic and screened for this study. Men and anatomical bladder outlet obstruction (anti‐incontinence procedures, urethral strictures or advanced pelvic organ prolapse) were excluded from the study. Severe debilitating MS, history of seizures, pregnancy or planning to become pregnant, contraindications to MRI, history of augmentation cystoplasty and presence of other neurological disorders besides MS were also excluded. Patients with active UTI can be treated and subsequently screened for the trial.

Twenty‐seven ambulatory female MS patients met the inclusion criteria and were divided into two groups based on their cystometric studies during their initial urodynamic study visit: patients without VD (denoted V, *n* = 14) who voided spontaneously and had a post‐void residual (PVR) volume of less than 40% of their bladder capacity (BC, defined as the sum of their PVR and voided volume) and patients with VD (denoted VD, *n* = 13) who performed self‐catheterization or had a PVR volume of 40% or more of the BC.^15^ All patients completed a detailed history, physical examination and validated questionnaires that included the Urogenital Distress Inventory (UDI‐6), Incontinence Impact Questionnaire 7 (IIQ‐7), Hamilton Anxiety Rating Scale (HAM‐A) and MRI Safety Screening Questionnaire.

### 2.2 Concurrent fMRI/UDS paradigm

The detailed protocols of the fMRI‐UDS examination in our MS and Healthy Control cohorts have been reported previously.^16^, ^17^ A Food and Drug Administration (FDA)‐approved, research‐dedicated 3‐T full‐body MRI scanner (Philips Ingenia) with standard 12‐channel head coil was used for the fMRI examinations.

Prior to the start of fMRI examination, subjects were asked to completely empty their bladder (spontaneously or via catheterization). Dual‐lumen 7‐Fr MRI‐compatible UDS bladder and rectal catheters were placed while the patients were in the scanner and the tubing was extended out of the MRI scanner room into the control room to record abdominal, vesical and detrusor pressures via a Laborie UDS machine.

Anatomical, DTI and fMRI images were obtained as in our previously established protocol.^16^, ^17^ During the concurrent fMRI/UDS, the bladder was gradually filled with room‐temperature sterile saline at 50 ml/min until subjects signalled a ‘strong desire to void’. Next, subjects were instructed to hold for 30 s, after which they were given permission to start voiding. If subjects were unable to void, the bladder was drained passively. After attempts to void were completed, the cycle was repeated three to four times depending on the patients' tolerance for the procedure. Care was taken not to exceed 45 min for the total duration of the fMRI examination.

### 2.3 FC quantification

AFNI (https://afni.nimh.nih.gov/) were used to identify significant brain activated regions (*p* < 0.05) at the time of voiding initiation using the generalized linear model (GLM) for each subject. Second‐level GLM analysis yielded significantly activated brain regions for each group. An average blood oxygenation level dependent (BOLD) activation map averaged over all subjects was created. The voiding initiation network (VIN) in MS was defined by including only the highest activated brain regions from the average BOLD activation map (*t*‐value > 3.5, *n* = 227) as previously reported.^18^ For each subject, the strength of FC of these brain regions in the VIN were quantified as FC values.

### 2.4 SC quantification

DTI images were aligned onto the ICBM‐DTI81 atlas,^19^ which consists of 50 white matter tracts, using AFNI. Fractional anisotropy (FA) value, which describes the main diffusion direction and refers to how well neuronal signals are transduced along the white matter tract, was obtained from all 50 tracts for each subject. FA was used as a metric for white matter tract integrity and SC.

### 2.5 ML analysis

Different ML algorithms may perform with varying success based on the hidden relationship in the data. Some algorithms are better suited for linear relationships; others are optimized for non‐linear relationships. Four ML algorithms [random forests (RF), neural networks (NN), generalized linear model (GLM) and partial least squares (PLS)],^12^ each based on a different principal algorithm, were therefore employed. FC values or SC values, alone or both, were investigated as predictor variables for the classification of subjects belonging to either the V or VD group.

The entire dataset was split into a training set (50%) and a test set (50%). Ten‐fold repeated cross validation with five repeats was used to train the ML algorithms. The algorithm with the highest area under the curve (AUC) of the receiver‐operating characteristic curve (ROC) was determined as the best‐performing algorithm. The predictor weights (ranging from 0 to 100) of FC or SC information of the best‐performing algorithm served as a measure to assess the relative importance of either information in predicting if a patient belonged in the V or VD group.

## 3 RESULTS

### 3.1 Subjects

Patients' demographics with p values from unpaired *t*‐tests and chi‐square test (alpha = 0.05) between V and VD groups are detailed in Table 1. No significant difference was observed between the two groups except for the two criteria that were used to define VD in the study population (self‐catheterization and %PVR/BC).

**TABLE 1:** Patient demographics of MS patients without and with VD (V and VD groups, respectively)

	Patients without VD *Mean (range)*	Patients with VD *Mean (range)*	*p*‐value
*Number of patients*	14	13	
Age, years	49.6 (37–66)	51.7 (33–85)	0.68
Body mass index, kg/m^2^	29.9 (20.8–43.5)	27.0 (20–37.4)	0.22
MS duration, years	15.1 (2–38)	14.8 (3–47)	0.95
UDI‐6	10.9 (2–24)	11.8 (6–21)	0.7
*Voiding pattern*			
Spontaneously voiding (%)	14/14 (100)	6/13 (46.2)	*5.95E‐03* *
Self‐catheterization (%)	0/14 (0)	9/13 (69.2)	*6.63E‐04* *
Number of deliveries	1.3 (0–3)	1.1 (0–4)	0.62
Previous hysterectomy (%)	3/14 (21.4)	1/13 (7.7)	0.64
Ambulatory (%)	14/14 (100)	13/13 (100)	N/A
Use of overactive bladder medication^a^	12/14 (85.7)	11/13 (84.6)	1.00
*UDS data*			
Bladder capacity, ml	412.6 (194–680)	368.6 (139–645)	0.50
Post‐void residual, ml	49.5 (0–150)	199.5 (58–370)	*9.60E‐05* *
%PVR/BC	13.1 (0–33.5)	53.7 (30.6–92.0)	*8.26E‐07* *
Detrusor sphincter dyssynergia (%)	1/14 (7.1)	3/13 (23.1)	0.53

*Note*: Unpaired *t*‐tests and chi‐square tests (alpha = 0.05) were performed to compare between the two groups.

^a^ Overactive bladder medication includes botulinum toxin‐A injection, solifenacin, mirabegron, oxybutynin or a combination of two of the aforementioned medications.

^*^ Significant difference (*p* < 0.05).

### 3.2 FC as predictors

**FIGURE 1:**
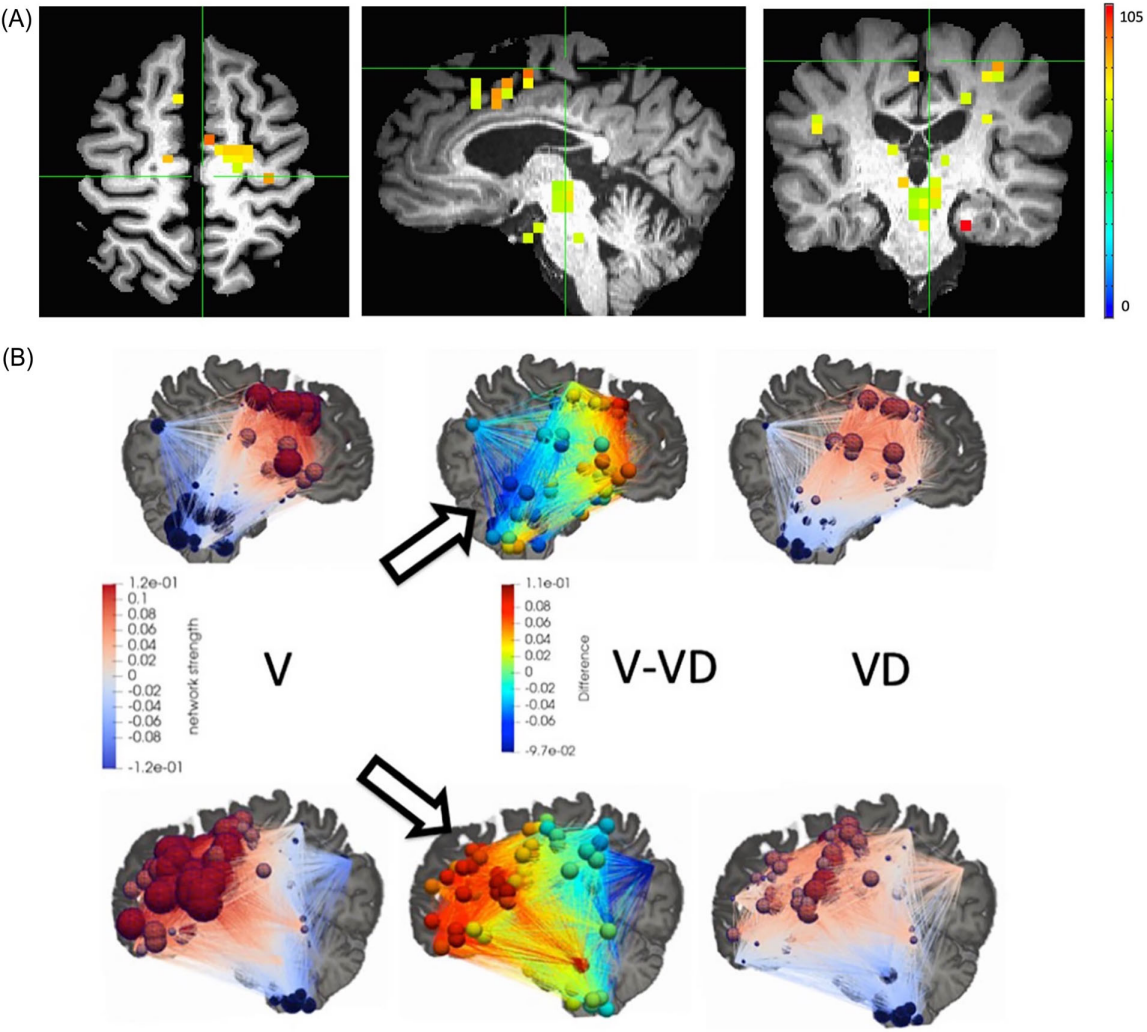
Classification of VD utilizing functional brain connectivity. (A) Brain regions with the most significant difference (*p* < 0.05) in FC strength between V and VD groups. Warmer colour denotes larger a difference. (B) Spheres denote regions that are part of the voiding initiation network (VIN), whose functional connectivity was used as predictor for patients who are voiders (V) or patients who have voiding dysfunction (VD). Strength of FC in the VIN in MS patients who void spontaneously (V, left) shows higher polarization (higher in the frontal brain regions and weaker in the cerebellar regions and the precuneus) versus voiding dysfunction (VD, right). Middle panel denotes difference in FC strength between V and VD. Arrows point to regions of polarization (frontal brain and cerebellum).

Dorsal vagal motor nuclei, pontine storage and micturition centre, periaqueductal grey (PAG), ventral tegmental area, substantia nigra, red nuclei, thalamus, cingulate, insula and cortical regions in the frontal, parietal and mesial temporal lobes were identified as part of the VIN. Using FC values as predictors, AUC values for RF, NNET, GLM and PLS were 0.86, 0.71, 0.53 and 0.89, respectively. Using the best‐performing algorithm (PLS), voiders showed a higher polarization of connectivity (higher in the frontal brain regions and weaker in the cerebellar regions and the precuneus) than VD subjects (Figure 1B). The most important brain regions identified for classification were the left frontal brain (middle, medial, inferior frontal gyrus) and left cingulate (Figure 1A), similar to results previously reported by our group.^12^

### 3.3 SC as predictors

**FIGURE 2:**
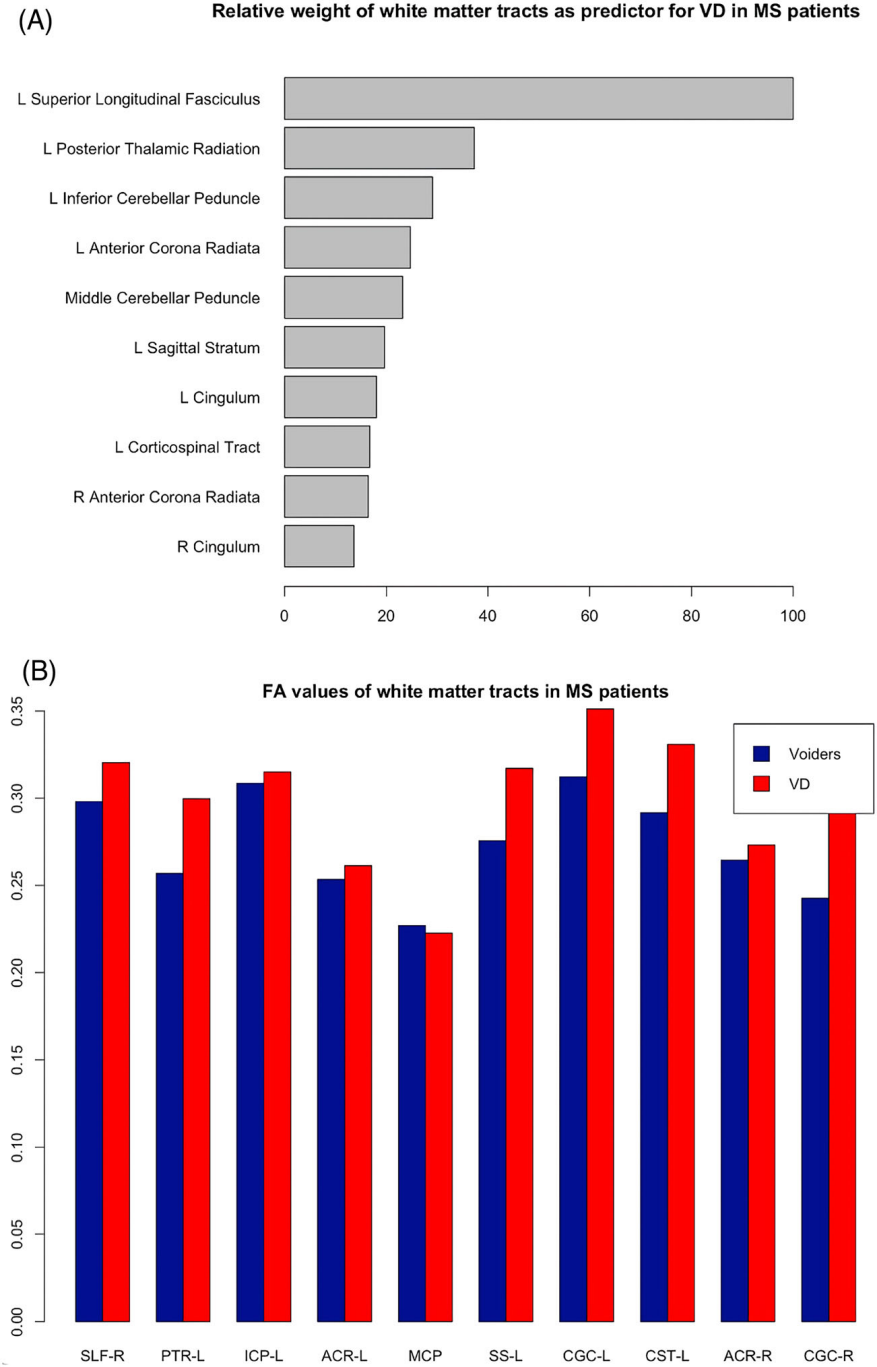
Classification of VD utilizing structural brain connectivity. (A) Relative weight of predictor of the 10 most important white matter tracts in predicting voiding dysfunction (VD) in female multiple sclerosis patients using the best‐performing algorithm (random forest). (B) Mean FA values of the 10 most important white matter tracts in predicting VD. ACR‐L, left anterior corona radiata; ACR‐R, right anterior corona radiata; CGC‐L, left cingulum; CGC‐R, right cingulum; CST‐L, left corticospinal tract; ICP‐L, left inferior cerebellar peduncle; MCP, middle cerebellar peduncle; PTR‐L, left posterior thalamic radiation; SLF‐R, right superior longitudinal fasciculus; SS‐L, left sagittal stratum

Utilizing FA values as predictors, AUC values for RF, NNET, GLM and PLS were 0.93, 0.58, 0.51 and 0.65, respectively. Using the best‐performing algorithm (RF), the most 10 important white matter tracts used in classification of V or VD patients were listed in Figure 2A. Mean FA values of these tracts were lower in the V group compared with VD (Figure 2B).

### 3.4 Both FC and SC as predictors

**FIGURE 3:**
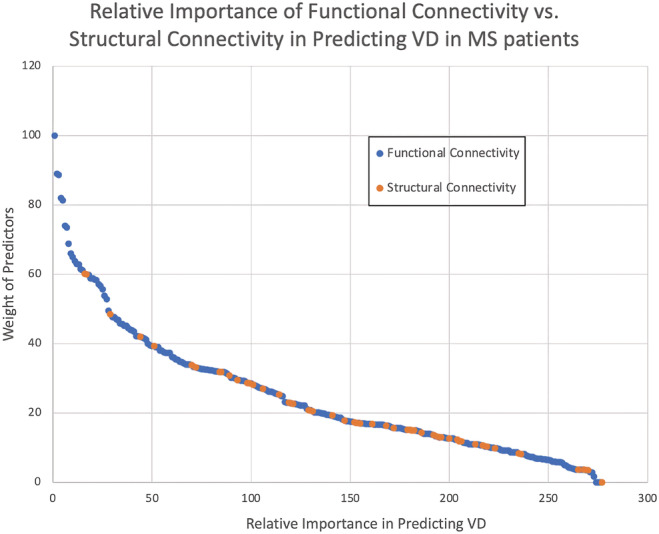
Relative importance of FC values (functional connectivity, blue) and FA values (structural connectivity, orange) in predicting voiding dysfunction in female multiple sclerosis patients using best‐performing (random forest) algorithm

When both FC and FA values were used as predictors, AUC values for RF, NNET, GLM and PLS were 0.96, 0.79, 0.83 and 0.95, respectively. FC values showed higher relative strength as predictors for classification of MS patients as V or VD, when compared with FA values (Figure 3). The 10 most important values for classification of V and VD were all FC values. Among white matter tracts, the most important one for classification of V and VD based on FA values was right superior longitudinal fasciculus, which was ranked 16th in relative importance of predictors.

## 4 DISCUSSION

The detrimental sequela of VD urges us to investigate means of early detection of VD in MS patients. Due to the demyelinating nature of MS, alternation of grey and white brain matters characteristics and connectivity might present differently in patients who have VD and those who do not. Previous studies have shown differences in both grey and white brain matter in MS patients with NLUTD who have VD compared with those who do not.^8^, ^20^, ^21^, ^22^ In this study, we demonstrate the ability of using ML to both classify and predict development of VD in MS patients using their neuroimaging information.

In grey matter areas, all MS individuals showed strong connectivity in the frontal brain regions while cerebellar regions exhibited reduced connectivity. This pattern is more pronounced in MS patients who void spontaneously, that is, stronger FC in the frontal lobes and higher disconnect in cerebellar areas, compared with those with VD. As an extension of our previous work,^12^ this result further affirms that patients with VD show more diffused FC patterns compared with those without. MS patients with a more severe disease course have also been shown to exhibit more divergent and distinguished FC patterns compared with those patients with less severe disease.^6^

The more diverging the pattern of FC is observed in MS patients, the higher the degree of severity and therefore possibility of developing VD symptoms in this cohort. Disruption of signal transduction from impaired white matter integrity in MS individuals with VD inhibits the formation of the FC pattern observed in MS patients who void spontaneously. Inhibition is most pronounced in the left frontal brain (including middle, medial and inferior frontal gyrus) and left cingulate, all of which have been shown to play a crucial role in bladder function in both healthy and MS patients.^18^, ^23^, ^24^

The 10 most important white matter tracts in classifying MS patients into V or VD groups have all been found in the literature to be involved in bladder function.^25^ Historically, FA describes the degree of anisotropy (‘spread’ in a direction) of a diffusion process. FA therefore can be used as a measure of integrity of white matter tract bundles, as higher FA values indicate coherent diffusion along the main tract direction (direction of signal transduction along white matter tract). Interestingly, mean FA values were higher in the VD group for almost all 10 tracts (except the middle cerebellar peduncle), indicating more preserved integrity of white matter in this group compared with those who void spontaneously. Although this is opposite of the expected trend, this result could indicate a compensatory mechanism in patients with VD as an effort to re‐establish connectivity in the presence of VD symptoms.^26^ This is further supported by the innate ability of the CNS to drive some remyelination of affected axons, however inefficient it may be.

When both grey (FC) and white (SC) matter information were combined as inputs for our ML algorithm to classify MS patients into V and VD groups, the 10 most important values established in predicting VD were all found to be FC, suggesting FC is more effective in predicting VD in MS patients. Consistent with results from using only white matter information, the right superior longitudinal fasciculus was found to be the most important white matter tract in predicting VD; however, it is ranked 16th in relative importance of predictors. Whereas SC are more persistent and stable, FC are more dynamic, fluctuating and highly time dependent.^5^ Higher importance of FC compared with SC in classifying VD in MS patients could suggest the susceptibility to change of FC due to establishment of new connections (neural plasticity) within the VIN in attempt to improve voiding function in MS patients.^27^ Even though white matter is affected in MS, new structural connections were likely formed to compensate for this loss and maintain normal voiding initiation, which was observed in the stronger FC in patients who void spontaneously.

Consistently for all three types of inputs (FC only, SC only, combined), the two best‐performing ML algorithms in predicting VD in MS patients were RF and PLS, whereas the worst‐performing algorithm was GLM. Because GLM is based on generalization of linear regression, this indicates the need for a complex non‐linear algorithm, which were offered by RF and PLS algorithms.^28^ The ML approach therefore offers the advantage of allowing access to the complex non‐linearity of the brain connectivity networks in classification of VD in MS patients, which is otherwise an impedance in other statistical methods.

## 5 CONCLUSIONS

To our knowledge, this is the first study to apply ML in predicting VD in MS patients using both grey (FC) and white (SC) brain matter information. Our study showed that ML is an effective method in classifying patients with MS into groups of those who void spontaneously or have VD based on their brain connectivity. Our results established FC as a better predictor for VD in female MS patients. This opens a new avenue for early symptom detection, prediction of disease progression and phenotyping for optimal treatment outcome, as well as provides new therapeutic targets in the treatment of VD in MS individuals. Additionally, this study sets the stage for utilizing non‐invasive neuroimaging techniques to predict LUTS in various aetiologies other than MS.

## CONFLICT OF INTEREST

All authors declare no conflict of interests.

## AUTHOR CONTRIBUTIONS

**Khue Tran**: Data curation, formal analysis, visualization, writing—original draft, writing—review and editing. **Betsy H. Salazar**: Formal analysis, validation, writing—original draft, writing—review and editing. **Timothy B. Boone**: Conceptualization, methodology, funding acquisition, supervision, writing—review and editing. **Rose Khavari**: Conceptualization, formal analysis, funding acquisition, investigation, project administration, methodology, project administration, supervision, validation, writing—review and editing. **Christof Karmonik**: Conceptualization, methodology, formal analysis, resources, software, supervision, visualization, writing—review and editing.
